# Radiomics for Tumor Characterization in Breast Cancer Patients: A Feasibility Study Comparing Contrast-Enhanced Mammography and Magnetic Resonance Imaging

**DOI:** 10.3390/diagnostics10070492

**Published:** 2020-07-18

**Authors:** Maria Adele Marino, Doris Leithner, Janice Sung, Daly Avendano, Elizabeth A. Morris, Katja Pinker, Maxine S. Jochelson

**Affiliations:** 1 Memorial Sloan Kettering Cancer Center, Department of Radiology, Breast Imaging Service, New York, NY 10065, USA; mariaadele.marino@gmail.com (M.A.M.); doris.leithner@gmail.com (D.L.); sungj@mskcc.org (J.S.); dlilahh1@gmail.com (D.A.); morrise@mskcc.org (E.A.M.); pinkerdk@mskcc.org (K.P.); 2 Department of Biomedical Sciences and Morphologic and Functional Imaging, University of Messina, 98100 Messina, Italy; 3 Department of Diagnostic and Interventional Radiology, University Hospital Frankfurt, 60590 Frankfurt, Germany; 4 Department Breast Imaging, Breast Cancer Center TecSalud, ITESM Monterrey, Monterrey 64718, Nuevo Leon, Mexico; 5 Department of Biomedical Imaging and Image-Guided Therapy, Division of Molecular and Gender Imaging, Medical University Vienna, 1090 Vienna, Austria

**Keywords:** radiomics, contrast-enhanced mammography, breast cancer, texture analysis, prognosis, diagnosis, characterization, magnetic resonance imaging

## Abstract

The aim of our intra-individual comparison study was to investigate and compare the potential of radiomics analysis of contrast-enhanced mammography (CEM) and dynamic contrast-enhanced magnetic resonance imaging (DCE-MRI) of the breast for the non-invasive assessment of tumor invasiveness, hormone receptor status, and tumor grade in patients with primary breast cancer. This retrospective study included 48 female patients with 49 biopsy-proven breast cancers who underwent pretreatment breast CEM and MRI. Radiomics analysis was performed by using MaZda software. Radiomics parameters were correlated with tumor histology (invasive vs. non-invasive), hormonal status (HR+ vs. HR−), and grading (low grade G1 + G2 vs. high grade G3). CEM radiomics analysis yielded classification accuracies of up to 92% for invasive vs. non-invasive breast cancers, 95.6% for HR+ vs. HR− breast cancers, and 77.8% for G1 + G2 vs. G3 invasive cancers. MRI radiomics analysis yielded classification accuracies of up to 90% for invasive vs. non-invasive breast cancers, 82.6% for HR+ vs. HR− breast cancers, and 77.8% for G1+G2 vs. G3 cancers. Preliminary results indicate a potential of both radiomics analysis of DCE-MRI and CEM for non-invasive assessment of tumor-invasiveness, hormone receptor status, and tumor grade. CEM may serve as an alternative to MRI if MRI is not available or contraindicated.

## 1. Introduction

Breast cancer is the most common cancer in women, accounting for 30% of all new cancer diagnoses in women [1]. Breast cancer prognosis is related to different factors, such as histopathologic subtypes, immunohistochemical (IHC) receptor status, and tumor grade [2,3,4], which are typically assessed with invasive tissue sampling. However, biopsies provide mere snapshots of tumor biology and are subject to site selection bias and thus cannot always provide characterization of the entire tumor. Radiomics analysis can automatically extract, quantify, and mine high-dimensional features that are imperceptible to human eyes from medical imaging data in a non-invasive and cost-effective way [5,6,7]. The central premise of radiomics analysis is that the extracted imaging features are representative of the phenotypic and genotypic processes of the entire tumor and therefore allow relevant insights into tumor biology [8,9]. Radiomics analysis can be coupled with the majority of clinically available medical imaging technologies, such as computed tomography, dynamic contrast enhanced magnetic resonance imaging (DCE-MRI), ultrasound, mammography, digital breast tomosynthesis, and positron emission tomography/computed tomography [10].

In breast imaging, DCE-MRI of the breast is the most sensitive test for breast cancer detection and characterization and has multiple established indications [11,12,13,14,15,16]. Similar to DCE-MRI, contrast-enhanced mammography (CEM) provides functional information on neoplastic neo-angiogenesis, thereby achieving improved sensitivity compared to mammography, especially in dense breasts [17], and providing staging information that is at least equivalent to DCE-MRI [18]. Therefore, CEM is an attractive alternative when MRI is not available, contraindicated, or poorly tolerated [19].

Several studies have already reported the value of radiomics analyses of DCE-MRI, including the value of DCE-MRI morphologic and functional radiomic features to provide insight into individual genomic signatures, breast cancer molecular subtypes, and clinically used recurrence scores [7,20,21,22,23]. There are fewer studies on the value of radiomics analyses of CEM; nevertheless, based on these limited studies, the results have been encouraging [23,24,25]. Recently, we reported preliminary results on the potential of CEM radiomics analysis of 100 patients in a larger-scale CEM-only study [25]. A subset of these patients had also undergone concurrent MRI, allowing us to perform an intra-patient comparison of the value of radiomics analysis for molecular subtyping of both techniques in this current study.

The aim of this study was to investigate and compare the potential of radiomics analysis of CEM and DCE-MRI of the breast for the non-invasive differentiation of tumor invasiveness, hormone receptor status, and tumor grade in patients with primary breast cancer.

## 2. Materials and Methods

In this HIPAA-compliant retrospective institutional review-board-approved( Code 12-214, Date 10/2012, Institutional Review Board Memorial Sloan Kettering Cancer Center) single-institution study, informed consent was waived.

### 2.1. Study Cohort

We included 118 patients over 21 years of age who underwent CEM and breast MRI between January 2010 and April 2018 as part of their inclusion in two prospective CEM studies. Sixty patients with breast cancer were part of a prospective study [18,26] that investigated the utility of CEM for breast cancer detection and staging. The remainder of the patients were participants of a prospective study in which CEM was compared with MRI for the screening of women with greater than 15% lifetime risk of developing breast cancer and who were diagnosed with breast cancer during the study. The prospective studies did not address any radiomics analysis performed in the current study. CEM and MRI were performed with a mean time of 5.8 days (range, 0–66 days) within each other, and only patients who underwent 3T breast MRI at our institution were included. All patients except one (*n* = 49, 98%) underwent MRI and CEM within one month of each other, with a mean of 5.8 days between the two examination. In one patient, MRI and CEM were performed 66 days apart from each other.

**Figure 1:**
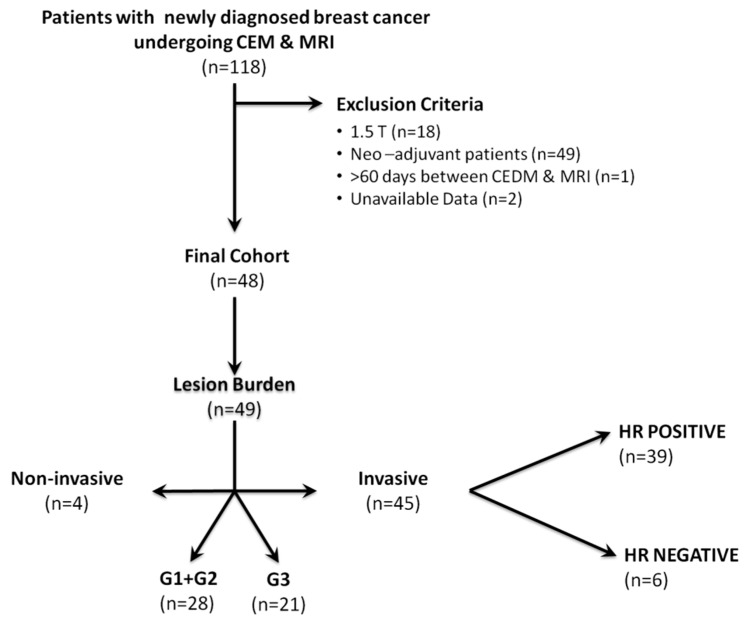
Details of lesions characteristics stratified by invasiveness, hormonal status, and grading. Abbreviations: CEM, contrast-enhanced mammography; MRI, magnetic resonance imaging; HR, hormone receptor; G1, Grade 1; G2, Grade 2; G3, Grade 3.

Sixty-nine patients were excluded for the following reasons: (a) different field strength of the MRI magnet (*n* = 18); (b) time interval between examinations was over 10 weeks (*n* = 1); (c) patients receiving neoadjuvant treatment (*n* = 49); and (e) data not available for retrospective analysis (*n* = 2). Figure 1 summarizes the flowchart of patient inclusion and the lesion burden of included patients.

### 2.2. Contrast-Enhanced Mammography Technique

The CEM technique has been previously described [26]. CEM was performed by using a GE SenoBright mammography unit. Patients were given 1.5 mL/kg body weight of Omnipaque 350 (iohexol, GE, Millwaukee, USA) (https://www.gehealthcare.com/products/contrast-media/omnipaque) through a 20-gauge needle, at an injection rate of 3 mL/s, with a maximum injected volume of 150 mL. Once contrast injection was complete, there was a 2.5–3 min delay during which time the patient was positioned for her mammogram. Mammographic imaging was then performed with almost simultaneous low-energy (26–30 kVp) and high-energy (45–49 kVp) images. Medio-lateral-oblique (MLO) and craniocaudal (CC) views of each breast were obtained and completed within 5 min of completion of contrast injection. The total examination time was approximately 8–9 min. The low-energy images which were generated were interpreted as the digital mammogram. A recombination algorithm subtracted out the unenhanced breast tissue and provided a subtracted image that highlighted areas of contrast enhancement.

### 2.3. Magnetic Resonance Imaging Technique

MRI examinations were performed with the patient prone on a 3.0-T commercially available system (General Electric Medical Systems, Milwaukee, WI, USA), using a dedicated 8-channel or 16-channel phase array breast coil. Integrated Parallel Acquisition Techniques (iPAT) were utilized for imaging both breasts simultaneously. The standard MRI examination included a localizing sequence followed by sagittal fat-suppressed and sagittal T1-weighted sequences.

Two DCE-MRI protocols were used for the post-contrast phases, sagittal and axial. In Table 1, DCE-MRI parameters for both sagittal and axial protocols are summarized. Unenhanced images were subtracted from the contrast-enhanced images on a pixel-by-pixel basis, producing subtracted post-contrast subtraction sequences. Maximum-intensity projection images were generated by utilizing the first post-contrast and the first post-contrast subtracted data. Gadoteratemeglumine (Gd-DOTA; Dotarem^®^, Guerbet, Roissy, France) was injected intravenously as a bolus (0.1 mmol/kg body weight), by a power injector (Spectris Solaris EP, Medrad, Pittsburgh, PA, USA), at 4 mL/s, followed by a 20 mL saline flush.

### 2.4. Segmentation of Lesions

**Figure 2:**
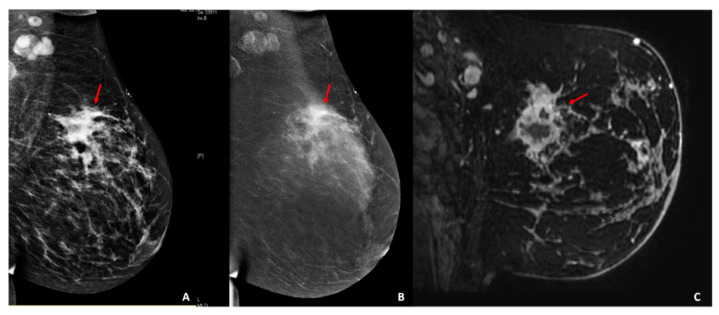
Palpable left abnormality in a 44-year-old patient. (**A**) Digital mammography and (**B**) contrast-enhanced mammography of the left breast, mediolateral-oblique view. (**A**,**B**) The breast shows scattered fibroglandular tissue. Minimal background parenchymal enhancement is present. In the left upper-outer quadrant, there are multiple masses with associated focal asymmetry and suspicious enhancement extending anteriorly toward the nipple (red arrows). (**C**) Sagittal fat-suppressed T1-weighted image acquired after the administration of intravenous gadolinium (2 min) twelve days after the contrast-enhanced mammography examination. There is abnormal enhancement extending from the 12/1 o’clock axis posteriorly to the nipple, with multiple areas of enhancement (red arrow). Pathology results yielded invasive triple-negative ductal carcinoma, with positive axillary lymph-nodes.

**Figure 3:**
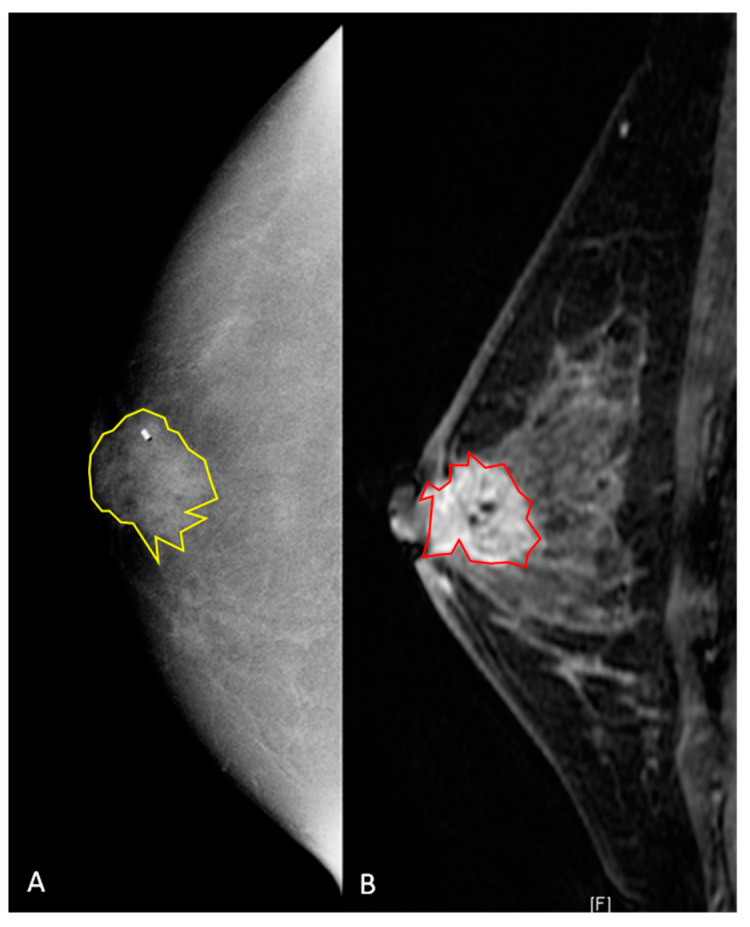
A 42-year old women with a biopsy-proven invasive ductal carcinoma (G3, HR positive, HER2 negative) in the right retro-areolar space. (**A**) Contrast-enhanced mammography shows, on the right, a 22 mm rounded area of enhancement. The lesion was manually segmented (2D yellow region of interest), and the clip marker had to be included in the segmented area. (**B**) Sagittal fat-saturated post-contrast enhanced T1-weighted image. The breast is heterogeneously dense with moderate background enhancement. In the retroareolar right breast, there is a 22 mm spiculated mass containing a localizing clip from biopsy. The mass is inseparable from the nipple, which appears slightly retracted, and there is associated skin thickening. The lesion was manually segmented (2D red region of interest).

All DICOM (Digital Imaging and Communications in Medicine) images were transferred to a database and loaded into the open-source image processing tool OsiriX (OsiriX Foundation, Geneva, Switzerland). Lesion segmentation was performed on a per-patient basis, and in patients with multifocal disease, the lesion with the largest diameter was selected (Figure 2 and Figure 3). For texture analysis, a fellowship-trained radiologist with over 4 years of experience in breast imaging manually delineated the borders of the lesions for both CEM and DCE-MRI DICOM images, including all the enhancing parts of the index cancer, excluding foreign bodies (i.e., clips). For CEM DICOM images, the borders were delineated in either the CC or MLO view, depending on which view the tumor was most conspicuous. Radiomics analysis was performed by using MaZda software (Technical University of Lodz, Poland). Radiomic features were derived from the first-order histogram (HIS): co-occurrence matrix (COM), run-length matrix (RLM), absolute gradient (GRA), autoregressive model (ARM), the discrete Haar wavelet transform (WAV), and lesion geometry (GEO). In Supplementary Materials Table S1, all the specifics of the used radiomics features are listed. For further information about the radiomic features used, please refer to Szczypiński et al. [27].

### 2.5. Histopathology

Histopathology results, including grade and IHC status, were recorded from biopsy specimens and served as the reference standard. An Automated Ventana Benchmark XT device was used as standard protocol to evaluate estrogen receptor (ER), progesterone receptor (PR), and human epidermal growth factor receptor 2 (HER2) status according to current American Society of Clinical Oncology/United States and Canadian Academy of Pathology guidelines, with 1% staining being considered positive [28]. HER2 status of 0 or 1+ was considered negative, 2+ equivocal, and 3+ positive. Patients with equivocal HER2 were evaluated, using fluorescence in situ hybridization, and considered as positive if HER2 gene amplification was observed [29].

### 2.6. Statistical Analysis

Fisher, probability of error and average correlation (POE + ACC), and mutual information (MI) coefficients were used for feature selection. Linear discriminant analysis, followed by k-nearest neighbor classification (with leave-one-out cross-validation), was used for pairwise texture-based separation of tumor invasiveness/hormonal status/tumor grade. Radiomics parameters were correlated with tumor invasiveness according to histology (invasive vs. non-invasive), hormonal status (HR+ vs. HR−), and grading (low grade G1 + G2 vs. high grade G3). Inter-rater agreement of MRI fibroglandular tissue (FGT), and background parenchymal enhancement (BPE) in both CEM and MRI was assessed by using kappa statistics with ≤0 as indicating no agreement, 0.01–0.20 as none to slight, 0.21–0.40 as fair, 0.41–0.60 as moderate, 0.61–0.80 as substantial, and 0.81–1.00 as almost perfect agreement.

## 3. Results

### 3.1. Patient and Breast Cancer Characteristics

A total of 49 histopathologically verified breast cancers (mean size 24.7 ± 20 mm; range 4–87 mm) in 48 women (mean age, 50.7 ± 8 years; range, 38–74 years) were detected with CEM and MRI. 

In terms of tumor invasiveness, 4/49 (8%) breast cancers were purely non-invasive cancers (mean size 21.5 ± 15 mm, range 6–42 mm), and 45/49 (92%) were invasive cancers (mean size 25 ± 20.6 mm, range 4–87 mm). Among the invasive cancers, 42/45 (93%) were invasive ductal carcinomas (mean size 24.9 ± 20.9 mm, range 4–87 mm), and 3/45 (7%) were invasive lobular cancers (mean size 25.6 ± 18.7 mm, range 9–46 mm). In terms of HR status, among the 45 primary breast carcinomas, 39/45 (87%) were HR+, and 6 (13%) were HR−. Four breast cancers were HER2 positive. In terms of tumor grade, 5/49 (10%) breast cancers were G1 cancers (DCIS = 1, invasive cancer = 4); 23/49 (47%) were G2 cancers (DCIS = 2; invasive cancer = 21), and 21/49 (43%) were G3 cancers (DCIS = 1; invasive cancer = 20). Table 2 describes the histopathological diagnosis of all breast cancers included in the study, according to histopathological subtype.

### 3.2. Radiomics Results for CEM

The following accuracies were achieved for the differentiation of invasive and non-invasive breast cancers: Fisher, 92% (COM); POE, 90% (COM); and MI, 88%. The following accuracies were achieved for the differentiation of HR+ and HR− cancers: Fisher, 91.3% (WAV/RUN); POE, 95.6% (RUN); and MI, 86.8% (WAV + COM). The following accuracies were achieved for the differentiation of low grade (G1 + G2) and high grade G3 cancers: Fisher, 75.6% (WAV + RUN + COM); POE, 77.8% (RUN); and MI, 64.4% (WAV + COM) (Table 3).

### 3.3. Radiomics Results for MRI

The following accuracies were achieved for the differentiation of invasive and non-invasive breast cancers: Fisher, 90% (COM); POE, 88% (COM); and MI, 88% (COM). The following accuracies were achieved for the differentiation of HR+ and HR− cancers: Fisher, 76.1% (COM); POE, 80.4% (COM); and MI, 82.6% (COM). The following accuracies were achieved for the differentiation of low-grade (G1 + G2) and high-grade G3 cancers: Fisher, 77.8% (RUN); POE, 71.1% (COM); and MI, 73.3% (COM) (Table 3).

### 3.4. Inter-Rater Agreement

There was moderate inter-rater agreement for density assessed by using the BI-RADS density categories (k = 0.652). Substantial inter-rater agreement was reached (k = 0.738) for the assessment of fibroglandular tissue on breast MRI. BPE was calculated for both CEM and MRI. Substantial agreement was found for BPE on CEM (k = 0.629) and on MRI (k = 0.789).

## 4. Discussion

In breast cancer, relevant prognostic and predictive factors include receptor status, tumor grade, and invasiveness, which are usually assessed with invasive tissue sampling. However, a single biopsy of a potentially heterogenous tumor can provide a mere snapshot of tumor biology and is subject to site selection and sampling bias; thus, it may not be representative of tumor biology in its entirety. Therefore, there is a strong argument for the development of alternative and more comprehensive means to provide prognostic and predictive information that is derived from the tumor in its entirety. In this context, there is a unique opportunity for breast imaging coupled with radiomics analysis.

In this feasibility study that performed an intra-individual comparison of CEM and DCE-MRI, results indicate the potential of both CEM and DCE-MRI for the non-invasive differentiation of invasive and non-invasive breast cancers, HR+ and HR− breast cancers, and low-grade and high-grade breast tumors with similar high accuracies.

When conducting a radiomics analysis, Bhooshan et al. [30] found in their study that 353 MRI cases that DCE-MRI radiomics features were able to distinguish between non-invasive and invasive breast cancer types. In a radiomics analysis, Bhooshan et al. [31] also found that DCE-MRI was promising for distinguishing breast tumors of different grades. Different studies have reported significant correlations between imaging-enhancement kinetics from DCE-MRI and molecular breast cancer subtypes [22,32,33,34]. Mazurowski et al. [34] performed radiogenomic analysis on a subset of 48 cases and found an increased ratio of tumor to background parenchymal enhancement in the luminal B subtype, compared with other subtypes. Yamaguchi et al. [22] investigated the relationship between the kinetic curve pattern and molecular subtypes and showed that HR+/HER2− and triple-negative (TN) breast cancer subtypes demonstrated less washout on the delayed phase of enhancement compared with HER2–luminal (HR+/HER2+) and HER2-enriched (HR−/HER2+) subtypes. Blaschke et al. [33] reported that HER2+ tumors have a more rapid initial phase enhancement compared with other molecular subtypes. Li et al. [32] observed the faster contrast uptake in ER/PR−, and TN cancers relative to ER/PR+ and non-TN cancers. All of these studies indicate that enhancement kinetics with DCE-MRI may reflect underlying tumor biological characteristics.

To date, the evidence for radiomics using CEM is scarce, with only few published studies. Patel et al. [24] evaluated the use of a computer-aided diagnosis (CAD)–CEM in the diagnostic performance of CEM, compared with that of experienced radiologists. The authors constructed a predictive model by using a support vector machine (SVM) classification method with a set of both morphologic and textural features extracted from the low-energy and recombined images of 50 lesions. Based on the SVM classification, CAD-CEM achieved an overall diagnostic accuracy of 90% that outdid the diagnostic accuracy of the two radiologists, respectively of 78% and 86%. They therefore concluded that CAD-CEDM can provide complementary information to radiologists, mainly by reducing the number of false-positive findings. In a different study from the same group, Danala et al. [23] used the CAD scheme of CEDM images to classify breast masses. The authors used the segmentation results obtained from dual-energy subtracted images of a CEDM dataset of 111 breast lesions to build a multilayer perceptron machine learning system able to classify mass lesions. The use of CAD-CEDM in their study increased the accuracy in mass region segmentation.

While there is now a growing body of evidence that MRI and radiomics can add valuable information on tumor receptor status, grade, and molecular subtypes, data for CEM in this context are scarce. CEM is an emerging technology in breast imaging that, like MRI, combines a morphologic evaluation of the breast with an assessment of tumor neovascularity as an indicator of malignancy. CEM might therefore serve an alternative for patients who cannot undergo MRI. MR imaging is expensive and time-consuming and cannot be performed on all patients. Women who are claustrophobic and women with pacemakers or other implanted metallic materials cannot undergo breast MRI. In analogy to breast MRI, CEM is able to detect enhancing tumor vascularity after contrast administration with a sensitivity in detecting breast cancer of 96–100% [17]. Our group has recently demonstrated that there is a potential role for CEM and radiomics in the non-invasive differentiation of tumors with different degrees of invasiveness, hormone receptor status, and tumor grade [25], yet the question of whether the results are comparable to the more established technique, in this context, that is MRI, remained unanswered. In the current study, we investigated and performed an intra-individual comparison of the potential of radiomics analysis of CEM and DCE-MRI of the breast for the non-invasive differentiation of tumor invasiveness, hormone receptor status, and tumor grade in patients with primary breast cancer. Initial results indicate a potential for both techniques, with similar diagnostic accuracies.

We acknowledge some limitations of this study. The sample size is relatively small. It has to be noted that, in particular, that the number of HER2-positive breast cancers, with four cases, and pure non-invasive cancers, with four cases, is small; therefore, results, in this respect, are preliminary and have to be confirmed in larger studies. In addition, due to the small sample size, we did not divide our study population into a training dataset and a validation dataset, but used a k-nearest neighbor classifier with leave-one-out cross-validation, which does not require two separate datasets and which has been used successfully in numerous other studies [35,36,37]. Inherent to CEM technology, we could evaluate only tumor radiomics features from 2D images, which might not be as representative of tumor heterogeneity as a 3D volumetric assessment with DCE-MRI. However, despite this limitation, we achieved good results for the differentiation of tumors with different tumor receptor status, as well as invasiveness, indicating that valuable information can also be derived from 2D image analysis. All CEM and MRI examinations were performed with the same machine, at the same field strength, which assures standardized image acquisition, yet our results might not be equally applicable across vendor platforms.

In conclusion, preliminary results of this intra-individual comparison study indicate a potential of both radiomics analysis of DCE-MRI and CEM for non-invasive assessment of tumor-invasiveness, hormone receptor status, and tumor grade. CEM can therefore serve as an alternative when MRI is not available or there are contraindications for MRI and/or MRI contrast agents.

## Figures and Tables

**Table 1:** Dynamic contrast-enhanced (DCE)-MRI acquisition parameters.

Parameter	DCE-MRI (Sagittal)	DCE-MRI (Axial)
Sequence	3D T1w gradient echo VIBRANT	3D T1w gradient echo VIBRANT
Imaging plane	Sagittal	Axial
TR, ms	10	4.2
TE, ms	2.1	minimum
Flip angle, °	10	12
Number of excitations	1	1
Acquisition matrix	320 × 180 to 448 × 224	320 × 320 to 360 × 360
Reconstructed matrix	512 × 512	512 × 512
Field of view *, cm	20–25	32–38
Slice thickness, mm	3	1
Slice gap, mm	0	0
Number of slices	80–112	250–300
Fat suppression	On	On
Parallel imaging	–	ASSET
b-values, s/mm^2^	–	–
Time per frame, s	60	60
Number of phases	4 (1 pre- and 3 post-contrast)	4 (1 pre- and 3 post-contrast)
Acquisition time, min	8	8

Notes: 2D and 3D, two- and three-dimensional; VIBRANT, Volume Image Breast Assessment; T1w, T1-weighted; TR, repetition time; TE, echo time. * Field of view was a square with the side length specified in the table.

**Table 2:** Histopathological diagnosis of all malignant lesions according to histopathological subtype.

Histopathological Subtype	n	G1	G2	G3	HR+		HR−	
					HER2−	HER2+	HER2−	HER2+
DCIS	4 (8)	1 (25)	2 (50)	1 (25)	-	-	-	-
IDC	42 (86)	4 (10)	18 (42)	20 (48)	34 (81)	2 (5)	4 (9)	2 (5)
ILC	3 (6)	-	3 (100)	-	3 (100)	-	-	-

Notes: Numbers in parentheses represent percentages. Abbreviations: DCIS, ductal carcinoma in situ; HR, hormone receptor; IDC, invasive ductal carcinoma; ILC, invasive lobular carcinoma.

**Table 3:** Detailed results of group-wise radiomic feature-based cancer: diagnostic accuracies for radiomic features based on cancer invasiveness, hormone subtype, and grading for contrast-enhanced mammography (CEM) and dynamic contrast-enhanced magnetic resonance imaging (DCE-MRI).

Imaging Modality	*Cancer Subtype*			
		*Invasive Cancers*	*HR* *Positive ^a^*	*Low Grade*
**CEM**	*Non-Invasive* *Cancers*	6 best: 92% (COM/Fisher) 90% (COM/POE) 88% (COM/MI)	-	-
	*HR negative ^b^*	-	7 best: 91.3% (WAV-RUN/Fisher) 95.6% (RUN/POE) 86.8% (COM/MI)	-
	*High grade*	-	-	9 best: 75.6% (WAV + RUN + COM/Fisher) 77.8% (RUN/POE) 64.4% (WAV + COM/MI)
**MRI**	*Non-Invasive* *Cancers*	6 best: 90% (COM/Fisher) 88% (COM/POE) 88% (COM/MI)	-	-
	*HR* *negative ^b^*	-	6 best: 76.1% (COM/Fisher) 80.4% (COM/POE) 82.6% (COM/MI)	-
	*High grade*	-	-	6 best: 77.8% (RUN/Fisher) 71.1% (COM/POE) 73.3% (COM/MI)

Notes: ^a^ HR positive/HER2 negative or HR positive/HER2 positive. ^b^ HR negative/HER2 positive or HR negative/HER2 negative (triple negative). Abbreviations: MI, mutual information; POE, Probability of error.

## Supplementary Materials

The following are available online at https://www.mdpi.com/2075-4418/10/7/492/s1.

## References

[1] Siegel R.L., Miller K.D., Jemal A. (2019). Cancer statistics. CA Cancer J. Clin..

[2] Bedard P.L., Hansen A.R., Ratain M.J., Siu L.L. (2013). Tumour heterogeneity in the clinic. Nature.

[3] Li H., Zhu Y., Burnside E.S., Huang E., Drukker K., Hoadley K.A., Fan C., Conzen S.D., Zuley M., Net J.M. (2016). Quantitative MRI radiomics in the prediction of molecular classifications of breast cancer subtypes in the TCGA/TCIA data set. NPJ Breast Cancer.

[4] Bloom H.J., Richardson W.W. (1957). Histological grading and prognosis in breast cancer; a study of 1409 cases of which 359 have been followed for 15 years. Br. J. Cancer.

[5] Gillies R.J., Kinahan P.E., Hricak H. (2016). Radiomics: Images Are More than Pictures, They Are Data. Radiology.

[6] Parekh V., Jacobs M.A. (2016). Radiomics: A new application from established techniques. Expert Rev. Precis. Med. Drug Dev..

[7] Pinker K., Chin J., Melsaether A.N., Morris E.A., Moy L. (2018). Precision Medicine and Radiogenomics in Breast Cancer: New Approaches toward Diagnosis and Treatment. Radiology.

[8] Mazurowski M.A. (2015). Radiogenomics: What it is and why it is important. J. Am. Coll. Radiol. JACR.

[9] Saha A., Harowicz M.R., Mazurowski M.A. (2018). Breast cancer MRI radiomics: An overview of algorithmic features and impact of inter-reader variability in annotating tumors. Med. Phys..

[10] Valdora F., Houssami N., Rossi F., Calabrese M., Tagliafico A.S. (2018). Rapid review: Radiomics and breast cancer. Breast Cancer Res. Treat..

[11] D’Orsi C.J., Sickles E.A., Mendelson E.B., Morris E.A. (2013). ACR BI-RADS® Atlas.

[12] Sardanelli F., Boetes C., Borisch B., Decker T., Federico M., Gilbert F.J., Helbich T., Heywang-Köbrunner S.H., Kaiser W.A., Kerin M.J. (2010). Magnetic resonance imaging of the breast: Recommendations from the EUSOMA working group. Eur. J. Cancer.

[13] Mann R.M., Balleyguier C., Baltzer P.A., Bick U., Colin C., Cornford E., Evans A., Fallenberg E., Forrai G., Fuchsjäger M.H. (2015). Breast MRI: EUSOBI recommendations for women’s information. Eur. Radiol..

[14] Baltzer P.A.T., Kapetas P., Marino M.A., Clauser P. (2017). New diagnostic tools for breast cancer. Memo.

[15] Leithner D., Moy L., Morris E.A., Marino M.A., Helbich T.H., Pinker K. (2018). Abbreviated MRI of the Breast: Does It Provide Value?. J. Magn. Reson. Imaging JMRI.

[16] Marino M.A., Helbich T., Baltzer P., Pinker-Domenig K. (2018). Multiparametric MRI of the breast: A review. J. Magn. Reson. Imaging JMRI.

[17] Jochelson M. (2014). Contrast-Enhanced Digital Mammography. Radiol. Clin. N. Am..

[18] Jochelson M.S., Dershaw D.D., Sung J.S., Heerdt A.S., Thornton C., Moskowitz C.S., Ferrara J., Morris E.A. (2013). Bilateral contrast-enhanced dual-energy digital mammography: Feasibility and comparison with conventional digital mammography and MR imaging in women with known breast carcinoma. Radiology.

[19] James J.J., Tennant S.L. (2018). Contrast-enhanced spectral mammography (CESM). Clin. Radiol..

[20] Yamamoto S., Han W., Kim Y., Du L., Jamshidi N., Huang D., Kim J.H., Kuo M.D. (2015). Breast Cancer: Radiogenomic Biomarker Reveals Associations among Dynamic Contrast-enhanced MR Imaging, Long Noncoding RNA, and Metastasis. Radiology.

[21] Yamamoto S., Maki D.D., Korn R.L., Kuo M.D. (2012). Radiogenomic analysis of breast cancer using MRI: A preliminary study to define the landscape. AJR Am. J. Roentgenol..

[22] Yamaguchi K., Abe H., Newstead G.M., Egashira R., Nakazono T., Imaizumi T., Irie H. (2015). Intratumoral heterogeneity of the distribution of kinetic parameters in breast cancer: Comparison based on the molecular subtypes of invasive breast cancer. Breast Cancer (Tokyo).

[23] Leithner D., Horvat J.V., Marino M.A., Bernard-Davila B., Jochelson M.S., Ochoa-Albiztegui R.E., Martinez D.F., Morris E.A., Thakur S., Pinker K. (2019). Radiomic signatures with contrast-enhanced magnetic resonance imaging for the assessment of breast cancer receptor status and molecular subtypes: Initial results. Breast Cancer Res. BCR.

[24] Danala G., Patel B., Aghaei F., Heidari M., Li J., Wu T., Zheng B. (2018). Classification of Breast Masses Using a Computer-Aided Diagnosis Scheme of Contrast Enhanced Digital Mammograms. Ann. Biomed. Eng..

[25] Patel B.K., Ranjbar S., Wu T., Pockaj B.A., Li J., Zhang N., Lobbes M., Zhang B., Mitchell J.R. (2018). Computer-aided diagnosis of contrast-enhanced spectral mammography: A feasibility study. Eur. J. Radiol..

[26] Marino M.A., Pinker K., Leithner D., Sung J., Avendano D., Morris E.A., Jochelson M. (2019). Contrast-Enhanced Mammography and Radiomics Analysis for Noninvasive Breast Cancer Characterization: Initial Results. Mol. Imaging Biol..

[27] Jochelson M.S., Pinker K., Dershaw D.D., Hughes M., Gibbons G.F., Rahbar K., Robson M.E., Mangino D.A., Goldman D., Moskowitz C.S. (2017). Comparison of screening CEDM and MRI for women at increased risk for breast cancer: A pilot study. Eur. J. Radiol..

[28] Szczypiński P.M., Strzelecki M., Materka A., Klepaczko A. (2009). MaZda--a software package for image texture analysis. Comput. Methods Programs Biomed..

[29] Hammond M.E.H., Hayes D.F., Wolff A.C., Mangu P.B., Temin S. (2010). American society of clinical oncology/college of american pathologists guideline recommendations for immunohistochemical testing of estrogen and progesterone receptors in breast cancer. J. Oncol. Pract..

[30] Guiu S., Michiels S., André F., Cortes J., Denkert C., Di Leo A., Hennessy B.T., Sorlie T., Sotiriou C., Turner N. (2012). Molecular subclasses of breast cancer: How do we define them? The IMPAKT 2012 Working Group Statement. Ann. Oncol. J. Eur. Soc. Med. Oncol..

[31] Bhooshan N., Giger M.L., Jansen S.A., Li H., Lan L., Newstead G.M. (2010). Cancerous breast lesions on dynamic contrast-enhanced MR images: Computerized characterization for image-based prognostic markers. Radiology.

[32] Bhooshan N., Giger M., Edwards D., Yuan Y., Jansen S., Li H., Lan L., Sattar H., Newstead G. (2011). Computerized three-class classification of MRI-based prognostic markers for breast cancer. Phys. Med. Biol..

[33] Li H., Zhu Y., Burnside E.S., Drukker K., Hoadley K.A., Fan C., Conzen S.D., Whitman G.J., Sutton E.J., Net J.M. (2016). MR Imaging Radiomics Signatures for Predicting the Risk of Breast Cancer Recurrence as Given by Research Versions of MammaPrint, Oncotype DX, and PAM50 Gene Assays. Radiology.

[34] Blaschke E., Abe H. (2015). MRI phenotype of breast cancer: Kinetic assessment for molecular subtypes. J. Magn. Reson. Imaging JMRI.

[35] Mazurowski M.A., Zhang J., Grimm L.J., Yoon S.C., Silber J.I. (2014). Radiogenomic analysis of breast cancer: Luminal B molecular subtype is associated with enhancement dynamics at MR imaging. Radiology.

[36] Fruehwald-Pallamar J., Czerny C., Holzer-Fruehwald L., Nemec S.F., Mueller-Mang C., Weber M., Mayerhoefer M.E. (2013). Texture-based and diffusion-weighted discrimination of parotid gland lesions on MR images at 3.0 Tesla. NMR Biomed..

[37] Mayerhoefer M.E., Schima W., Trattnig S., Pinker K., Berger-Kulemann V., Ba-Ssalamah A. (2010). Texture-based classification of focal liver lesions on MRI at 3.0 Tesla: A feasibility study in cysts and hemangiomas. J. Magn. Reson. Imaging JMRI.

