# Supplementary material for: Radiomics for Tumor Characterization in Breast Cancer Patients: A Feasibility Study Comparing Contrast-Enhanced Mammography and Magnetic Resonance Imaging
Source: Diagnostics (Basel). 2020 Jul 18;10(7):492. doi: 10.3390/diagnostics10070492 (PMC7400681; doi:10.3390/diagnostics10070492)
Supplement: Supplementary file 1 [file diagnostics-10-00492-s001.pdf]

Supplementary Material

Table S1: Radiomic features from different categories

Lesion Geometry (GEO)	First-Order Histogram (HIS)	Absolute Gradient (GRA)	Run-Length Matrix (RLM)	Co-Occurrence Matrix (COM)	Autoregressive Model (ARM)	Wavelet Transform (WAV)
• Total number of geometric parameters: 73	• mean<br>• variance<br>• skewness<br>• kurtosis,<br>• 1-% percentile<br>• 10-% percentile<br>• 50-% percentile<br>• 90-% percentile<br>• 99-% percentile	• mean<br>• variance<br>• skewness<br>• kurtosis<br>• percentage of pixels with nonzero gradient.	• run length nonuniformity<br>• grey level nonuniformity<br>• long run emphasis<br>• short run emphasis<br>• fraction of image in runs	• angular second moment<br>• contrast<br>• correlation<br>• sum of squares<br>• inverse difference moment<br>• sum average<br>• sum variance<br>• sum entropy<br>• entropy<br>• difference variance<br>• difference entropy	• teta 1<br>• teta 2<br>• teta 3<br>• teta 4<br>• sigma	• WAVELET ENERGY

For further information on the features visit: http://www.eletel.p.lodz.pl/programy/mazda/download/FeaturerList.pdf
